# Respiratory Trajectories in Type 2 and 3 Spinal Muscular Atrophy in the iSMAC Cohort Study

**DOI:** 10.1212/WNL.0000000000011051

**Published:** 2021-01-26

**Authors:** Federica Trucco, Deborah Ridout, Mariacristina Scoto, Giorgia Coratti, Marion L. Main, Robert Muni Lofra, Anna G. Mayhew, Jacqueline Montes, Marika Pane, Valeria Sansone, Emilio Albamonte, Adele D'Amico, Enrico Bertini, Sonia Messina, Claudio Bruno, Deepak Parasuraman, Anne-Marie Childs, Vasantha Gowda, Tracey Willis, Min Ong, Chiara Marini-Bettolo, Darryl C. De Vivo, Basil T. Darras, John Day, Elizabeth A. Kichula, Oscar H. Mayer, Aledie A. Navas Nazario, Richard S. Finkel, Eugenio Mercuri, Francesco Muntoni

**Affiliations:** From the Dubowitz Neuromuscular Centre (F.T., M.S., M.L.M., F.M.) and Population, Policy and Practice Programme (D.R.), UCL GOS Institute of Child Health, London, UK; DINOGMI, University of Genoa (F.T.), IRCCS Istituto G. Gaslini, Italy; NIHR Great Ormond Street Hospital Biomedical Research Centre (D.R., F.M.), Great Ormond Street Institute of Child Health, University College London, and Great Ormond Street Hospital Trust, UK; Paediatric Neurology (G.C., M.P., E.M.), Catholic University; Centro Clinico Nemo (G.C., M.P., E.M.), Fondazione Policlinico Universitario Agostino Gemelli IRCSS, Rome, Italy; John Walton Muscular Dystrophy Research Centre (R.M.L., C.M.-B.), Newcastle University and Newcastle Hospitals NHS Foundation Trust, Newcastle Upon Tyne, UK; Departments of Neurology and Pediatrics (J.M., D.C.D.V.) and Departments of Rehabilitation and Regenerative Medicine (J.M.), Columbia University Irving Medical Center, New York, NY; Paediatric Neurology and Centro Clinico Nemo (V.S., E.A.), Milan; Unit of Neuromuscular and Neurodegenerative Disorders (A.D., E.B.), Post-Graduate Bambino Gesù Children's Research Hospital, IRCCS, Rome; Department of Clinical and Experimental Medicine (S.M.), University of Messina Paediatric Neurology and Nemo Sud Clinical Centre; Center of Translational and Experimental Myology (C.B.), IRCCS Istituto Giannina Gaslini, Genova, Italy; University Hospitals Birmingham NHSFT (D.P.); Leeds Children Hospital (A.-M.C.); Evelina Children's Hospital (V.G.), London; The Robert Jones and Agnes Hunt Orthopaedic Hospital (T.W.), Oswestry; Sheffield Children's Hospital (M.O.), UK; Department of Neurology (B.T.D.), Boston Children's Hospital and Harvard Medical School, MA; Stanford University (J.D.), Medical Centre, Palo Alto, CA; Divisions of Pediatric Neurology (E.A.K.), Pulmonology (O.H.M.) and Physical Therapy (A.M.G.), The Children's Hospital of Philadelphia, and the Perelman School of Medicine at the University of Pennsylvania, Philadelphia; and Divisions of Neurology (R.S.F.) and Pulmonary Medicine (A.A.N.N.), Department of Pediatrics, Nemours Children's Hospital, Orlando, FL.

## Abstract

**Objective:**

To describe the respiratory trajectories and their correlation with motor function in an international pediatric cohort of patients with type 2 and nonambulant type 3 spinal muscular atrophy (SMA).

**Methods:**

This was an 8-year retrospective observational study of patients in the International SMA Consortium (iSMAc) natural history study. We retrieved anthropometrics, forced vital capacity (FVC) absolute, FVC percent predicted (FVC%P), and noninvasive ventilation (NIV) requirement. Hammersmith Functional Motor Scale (HFMS) and revised Performance of Upper Limb (RULM) scores were correlated with respiratory function. We excluded patients in interventional clinical trials and on nusinersen commercial therapy.

**Results:**

There were 437 patients with SMA: 348 with type 2 and 89 with nonambulant type 3. Mean age at first visit was 6.9 (±4.4) and 11.1 (±4) years. In SMA type 2, FVC%P declined by 4.2%/y from 5 to 13 years, followed by a slower decline (1.0%/y). In type 3, FVC%P declined by 6.3%/y between 8 and 13 years, followed by a slower decline (0.9%/y). Thirty-nine percent with SMA type 2% and 9% with type 3 required NIV at a median age 5.0 (1.8–16.6) and 15.1 (13.8–16.3) years. Eighty-four percent with SMA type 2% and 80% with type 3 had scoliosis; 54% and 46% required surgery, which did not significantly affect respiratory decline. FVC%P positively correlated with HFMS and RULM scores in both subtypes.

**Conclusions:**

In SMA type 2 and nonambulant type 3, lung function declines differently, with a common leveling after age 13 years. Lung and motor function correlated in both subtypes. Our data further define the milder SMA phenotypes and provide information to benchmark the long-term efficacy of new treatments for SMA.

Spinal muscular atrophy (SMA) is an autosomal recessive neurodegenerative disorder characterized by progressive muscle wasting due to motor neuron degeneration, secondary to mutations in the survival motor neuron 1 (*SMN1*) gene.^1^ SMA is classified according to age at onset and maximal motor functional status achieved: weak infants unable to sit unsupported (type 1), nonambulant patients (type 2), and ambulant patients with childhood (type 3) and adult (type 4) onset.^2,3^

Respiratory impairment is the most frequent nonneurologic complication and the leading cause of mortality in SMA.^4^ Patients with SMA present with variable severity of chest wall distortion, paradoxical breathing, and impaired airway clearance and cough, compounded by bulbar muscle weakness.^5,6^ The assessment of respiratory function has gained interest in infants with SMA type 1^7,8^ because recently available treatments have improved patients' motor function and life expectancy.^9–11^ In contrast, very few studies have focused on the long-term respiratory progression in SMA types 2 and 3.^12–14^ The correlation between respiratory and motor function in these milder subtypes is interesting in light of the new therapeutic options.^15–18^ Intrathecal nusinersen and adeno-associated viral vector gene replacement therapy are commercially available (the latter currently only for patients <2 years of age). Both small molecules orally administered (NCT02908685)^19^ and intrathecal gene replacement (NCT03381729) are currently in clinical trials.

The aim of our work is to describe the respiratory features of pediatric patients with SMA type 2 and nonambulant SMA type 3 and their correlation with motor function in a multicenter international cohort.

## Methods

### Standard Protocol Approvals, Registrations, and Patient Consents

The study was approved by the Institutional Review Board (Ethics Committee) at each participating study site. Written informed consent was obtained from all participants (or guardians of participants) in the study (consent for research).

### Study Population

This was an 8-year (June 2010–September 2018) retrospective observational study of pediatric patients with SMA type 2 and nonambulant SMA type 3 (age <18 years). The data used in this study are part of the International SMA Consortium (iSMAc)^20^ composed of the SMA REACH-UK (NCT03520179), Italian SMA, and US Pediatric Neuromuscular Clinical Research Networks and additional centers: UK SMARTNET and C. Mondino and C. Besta Neurological Institutes (Italy).

Patients with SMA type 2 were classified as sitters and nonsitters (those who lost the ability to sit unsupported) at each recorded visit. Only patients with nonambulant SMA type 3 were included in this study in order to compare homogeneous respiratory trajectories not affected by changes in ambulatory status.

We subsequently excluded patients recruited in any interventional clinical trials or receiving nusinersen or onasemnogene abeparvovec, either commercially available or within the Expanded Access Programme. *SMN1* gene mutations and *SMN2* copies were recorded. Anthropometrics were collected. Either arm span or recumbent or ulnar length was used as a surrogate for height in forced vital capacity (FVC) percent predicted (FVC%P) calculation.^21^ Comorbid conditions affecting lung function such as aspiration, identified either clinically or by videofluoroscopy, were collected. Patients' nutritional status was postulated by body mass index (BMI) expressed as kilograms per meter squared. Patients' feeding status (oral nutrition, nasogastric tube or gastrostomy) was recorded. Scoliosis was defined as Cobb angle >10°. Scoliosis surgery technique was collected.

### Respiratory Function

Spirometry was performed at each site by either physiotherapists or respiratory physiologists who had received appropriate training and certification in the context of clinical trials. The best of 3 efforts deemed reliable by the operator was recorded according to international guidelines.^22^ FVC absolute (liters), FVC%P, peak expiratory flow (PEF) absolute (liters per minute), and PEF percent predicted (PEF%P) with the patient tested in a sitting position were collected.

Ventilation requirement, either noninvasive (NIV) or invasive (tracheostomy), and the use of assisted airway clearance were recorded.

### Motor Function

Motor function outcomes, namely Hammersmith Functional Motor Scale (HFMS) and revised Performance of Upper Limb (RULM) scores, were collected. HFMS was developed to assess the physical abilities of SMA type 2 and type 3 with limited ambulation. It is composed of 20 items with a maximal score of 40.^23^ The Upper Limb Module (ULM) was the first tool to assess the upper limb function in nonambulant SMA. It was created and validated in 2011 for nonambulant children from 30 months of age to adults.^24^ In 2017, ULM was critically re-evaluated, and the RULM was developed to tackle the ceiling effect observed with the ULM in nonambulant patients. RULM detects changes in upper limb function in a wide spectrum of weak and strong SMA. The RULM has a total of 19 items plus an entry item not included in the total score that serves as functional class identification. Its maximum total score is 37.^25^ A higher score on the HFMS, ULM, and RULM represents a higher level of function.

### Statistical Analysis

The primary outcome was the annual variation of FVC%P in SMA type 2 and nonambulant type 3 patients. Secondary outcome was the correlation between respiratory (FVC%P) and motor function (HFMS and/or RULM). The FVC%P trajectories before and after scoliosis surgery and the annual variations of FVC absolute, PEF%P, and PEF absolute were also analyzed.

The population characteristics are presented as mean (SD), median (range or interquartile range) for skewed data, and frequency (percentage) for categorical data.

For FVC%P, FVC absolute, and PEF%P, we estimated the mean annual change using mixed-effects regression models, accounting for the longitudinal data and age at baseline. Results are presented as mean annual change, or difference in mean annual change between subgroups, with 95% confidence intervals (CIs). Because the change in these outcomes was not linear over the full age range, we used linear splines to estimate and compare the relationships before and after 8 (SMA type 3) and 13 years of age. Using Kaplan-Meier and Cox regression analyses, we estimated the median age when FVC%P fell below 60%, 40%, and 20%; scoliosis surgery occurred; and gastrostomy was placed.

Correlation between respiratory (FVC%P) and motor function (HMFS and/or RULM score) was performed for both patients with SMA type 2 and those with type 3 by Spearman rank correlation. We reported the correlation between FVC%P and each motor functional score at the first available visit because the correlation factor was not different from that obtained when correlating FVC%P and motor function throughout the study period.

All analyses were conducted in Stata version 15 (StataCorp, College Station, TX) with a significance level of *p* < 0.05.

### Data Availability

The data that support the findings of this study are owned by the iSMAC academic consortium and available from the corresponding author on reasonable request.

## Results

### Study Population

**Figure 1:**
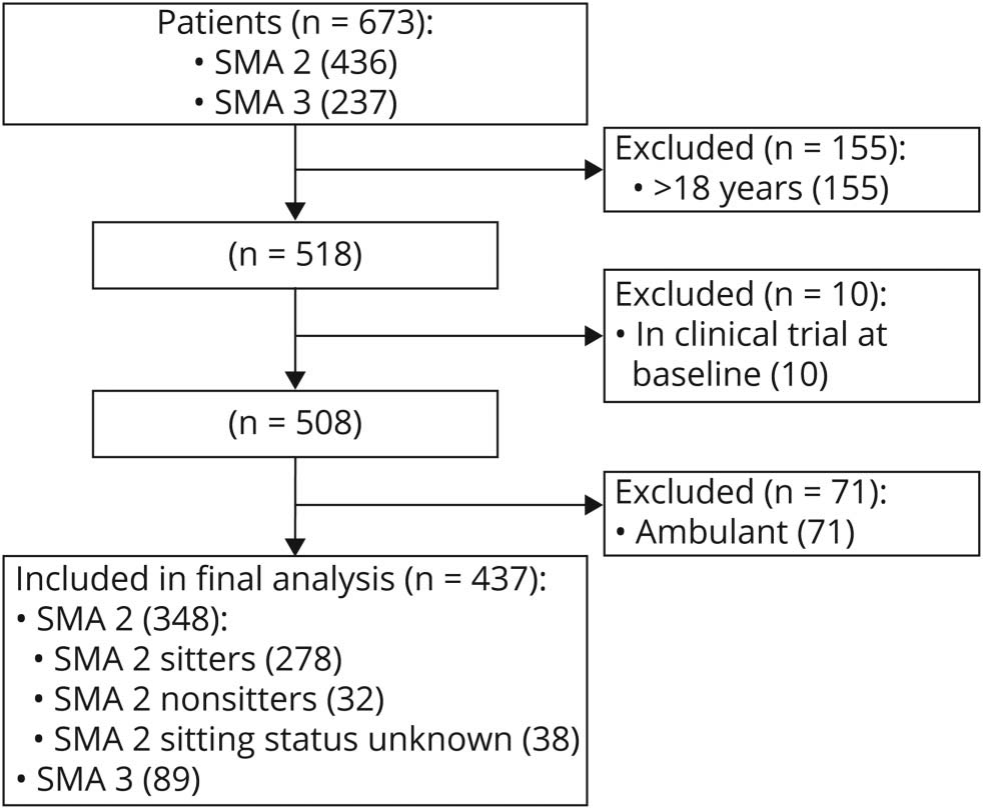
CONSORT Flowchart of Patients Included in the Final Analysis According to Inclusion and Exclusion Criteria Data were available for 673 patients in the whole cohort. Patients >18 years of age, patients enrolled in interventional clinical trial, and patients with spinal muscle atrophy (SMA) type 3 ambulant at the first recorded visit were excluded. The breakdown of patients (n = 437) included in the analysis refers to first visit. CONSORT = Consolidated Standards of Reporting Trials.

Data were available for 437 patients. There were 348 (80%) with SMA type 2. At first visit, 278 were sitters and 32 were nonsitters; sitting status was not available in 38. Fourteen patients who were sitters at the first visit lost their ability to sit independently during the follow-up. Eighty-nine (20%) had nonambulant SMA type 3 (figure 1). In both SMA type 2 subgroups, most patients had 3 copies of *SMN2*: they accounted for 89% and 67% of the available data in sitters and nonsitters, respectively. Patients with 2 copies of *SMN2* accounted for 9% and 33% of the available data in sitters and nonsitters.

Mean age at first visit was 6.9 (±4.4) years for SMA type 2 and 11.1 (±4) years for SMA type 3. Median follow-up was 1.2 years (interquartile range [IQR] 0–3.3 years, range 0–12.5 years).

Median BMI at first visit was 15.8 (14.0–19.1) kg/m^2^ in SMA type 2 and 18.1 (16.6–22.0) kg/m^2^ in type 3. BMI and FVC absolute at first visit positively correlated in both SMA type 2 (*r* = 0.5, *p* < 0.05) and type 3 (*r* = 0.6, *p* < 0.01). Throughout the study period, 9 patients with SMA type 2 and none of the patients with SMA type 3 required nasogastric tube. Only patients with SMA type 2 required gastrostomy (62/278), 25% of them by 12 years of age (table 1 and efigure 1).

**Table 1:**
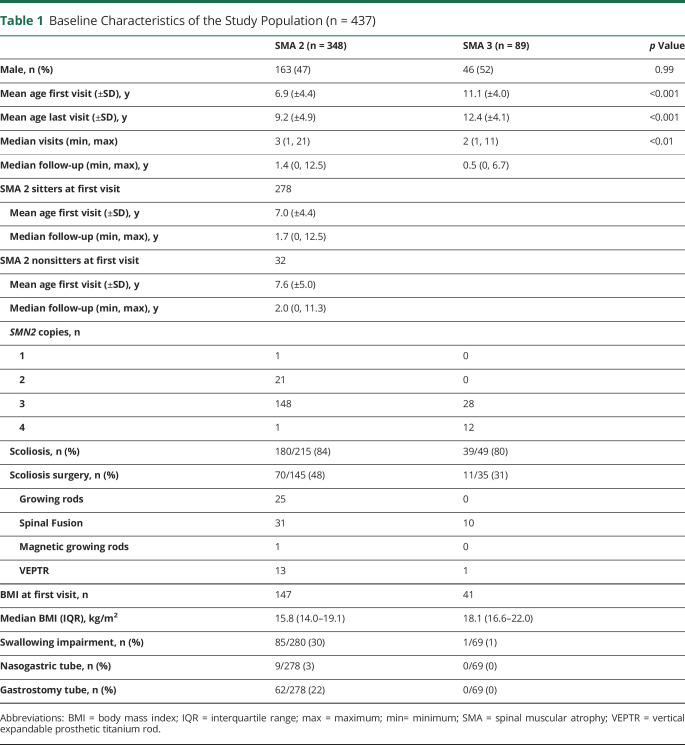
Baseline Characteristics of the Study Population (n = 437)

### Respiratory Progression and Respiratory Support

Data on FVC%P were available for 260 patients. Over the 8-year observation, the annual rate of decline of FVC%P between 5 and 18 years was 3.6% in SMA type 2 (95% CI −4.2% to −2.9%) and 3.5% in SMA type 3 (95% CI −5.6% to −1.4%). However, the trajectory of FVC%P progressed differently in the 2 SMA subgroups in the age range of 5 to 13 years, followed by a similar plateau phase after the age of 13 years.

Indeed, in SMA type 2, FVC%P (n = 200) declined by 4.2%/y (95% CI −4.8%/y to −3.7%/y) from 5 to 13 years of age, followed by a significantly slower (*p* < 0.001) decline of 1.0%/y (95% CI −2.1%/y to 0.2%/y). When SMA type 2 was subdivided into sitters and nonsitters, SMA type 2 sitters (n = 165) had an annual decline of FVC%P of 4.1% (95% CI −4.7% to −3.5%) from 5 to 13 years, followed by a slower (*p* < 0.001) progression of 1.3% (95% CI −2.5% to −0.04%). A similar decline (*p* = 0.15 vs sitters, adjusted for age) was observed in SMA type 2 nonsitters (n = 17). Their FVC%P from 5 to 13 years declined annually by 6.0% (95% CI −8.0% to −4.0%), followed by a slower progression.

**Figure 2:**
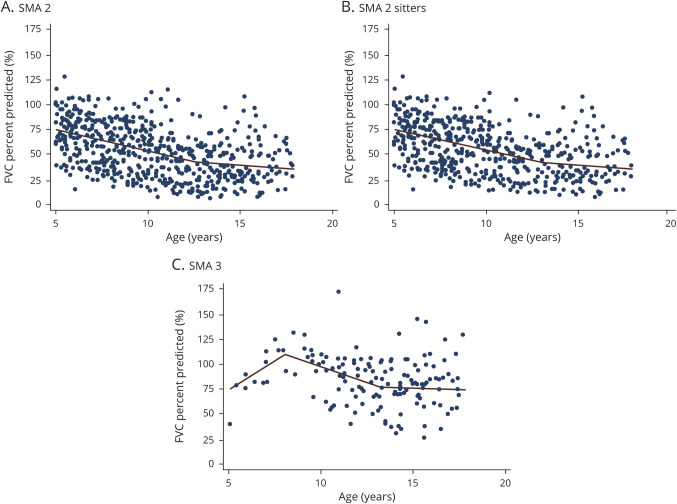
Rate of Decline of FVC%P in SMA Type 2, SMA Type 2 Sitters, and SMA Type 3 (A) Spinal muscle atrophy (SMA) type 2 (632 observations from 200 patients). The slope of forced vital capacity (FVC) percent predicted (FVC%P) at age 5 to 13 years was −4.2 (95% confidence interval [CI] −4.8 to −3.7, *p* < 0.01) and after age 13 years was −1.0 (95% CI −2.1 to 0.2, *p* = 0.1). The 2 slopes were significantly different (*p* < 0.001). (B) SMA type 2 sitters (565 observation from 165 patients). The slope of FVC%P at age 5 to 13 years was −4.1%/y (95% CI −4.7 to −3.5, *p* < 0.001) and after age 13 years was −1.3 (95% CI −2.5 to −0.04, *p* = 0.04). The 2 slopes were significantly different (*p* < 0.001). (C) SMA type 3 nonambulant (151 observations from 59 patients). FVC%P improved mildly from age 5 to 8 years by 11.8 (95% CI 4.5–19.1, *p* = 0.002) before declining from age 8 to 13 years by 6.3 (95% CI −8.7 to −3.8, *p* < 0.001). After age 13 years, FVC%P slope declined by 0.9 (95% CI −3.1 to 1.4, *p* = 0.46). The slopes for 5 to 8 and 8 to 13 years were significantly different (*p* < 0.001 and *p* < 0.01).

Nonambulant SMA type 3 (n = 59) had a 3-phase respiratory progression characterized by a mild increase of FVC%P from 5 to 8 years, followed by a steeper decline from 8 to 13 years and a leveling thereafter. In detail, in the age range of 5 to 8 years, FVC%P increased annually by 11.8% (95% CI (4.5%–19.1%); from age 8 to 13 years, FVC%P declined by 6.3% (95% CI −8.7% to −3.8%); and after 13 years, FVC%P declined significantly more slowly (*p* = 0.01) by 0.9% (95% CI −3.1% to 1.4%) (figure 2). The mild improvement in pulmonary function observed in the age range of 5 to 8 years is interesting and is reminiscent of improvement noticed in other outcome measures, although firm conclusions cannot be drawn due to the small sample size in that age window.

After 13 years of age, FVC%P stabilized, and its annual decline became similar in SMA type 2 sitters, nonsitters, and those with nonambulant SMA type 3. However, the estimated FVC%P was higher in SMA type 3 (65.8%, 95% CI 68.2%–83.4%) than in SMA type 2 sitters (41.0%, 95% CI 35.2%–46.9%) (*p* < 0.001) and nonsitters (32.1%, 95% CI 14.1%–50.1%) (*p* < 0.001). No differences were found between SMA type 2 sitters and nonsitters (*p* = 0.36).

**Figure 3:**
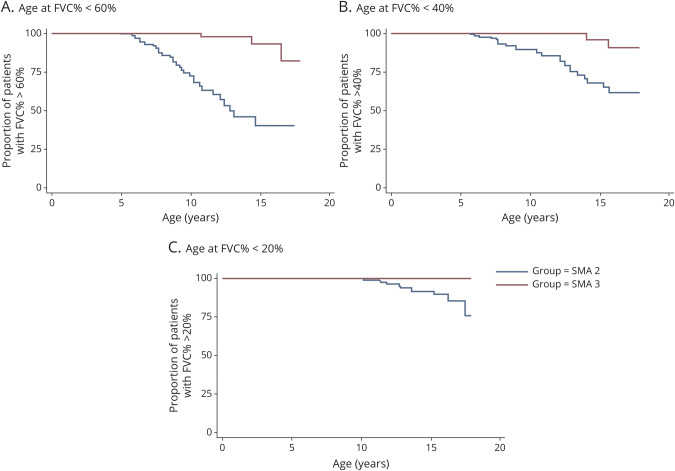
Age at Clinically Meaningful Thresholds of FVC%P (60%, 40%, 20%) in SMA Type 2 and 3. (A) At age 9.5 years, 25% of patients with spinal muscle atrophy (SMA) type 2 had forced vital capacity percent predicted (FVC%P) below 60%. Median (50%) age at FVC%P <60% was 12.8 years for SMA type 2. Fewer than 25% of patients with SMA type 3 had FVC%P below 60% (*p* < 0.001). (B) At age 13.4 years, 25% of patients with SMA type 2 and <20% of patients with SMA type 3 had FVC%P below 40% predicted (*p* < 0.01). (C) Fewer than 25% of patients with SMA type 2 and none of those with SMA type 3 had FVC%P below 20%.

Three significant lung function thresholds were evaluated in relation to the increased risk of sleep disordered breathing (FVC%P <60% and FVC%P <40%) and the development of diurnal respiratory failure (FVC%P <20%). One hundred eleven patients with SMA type 2 and 51 with type 3 had FVC%P >60%. The median (50%) age at FVC%P <60% was 12.8 years in patients with SMA type 2, while in <25% with SMA type 3, FVC%P fell below 60% (*p* < 0.001). One hundred fifty patients with SMA type 2 and 58 with SMA type 3 had FVC%P >40% at their first visit. At the age of 13.4 years, 25% with SMA type 2 had FVC%P <40%, while in those with SMA type 3, FVC%P fell below 40% in <25% (*p* < 0.01). Fewer than 25% of the 189 with SMA type 2 and none of the 60 with SMA type 3 in our cohort reached FVC%P <20% (figure 3).

Absolute FVC in SMA type 2 increased by 0.03 L/y (95% CI 0.02–0.05) with a stability between 10 and 14 years. There was no difference between SMA type 2 sitters and nonsitters (*p* = 0.54). In SMA type 3, absolute FVC steadily increased from age 5 to 18 years by 0.10 L/y (95% CI 0.04–0.16) (efigure 2).

Absolute PEF and PEF%P trajectories were available only in SMA type 2 (n = 63). PEF%P annually declined from 5 years of age by 4.1% (95% CI −6.2% to −1.9%). Absolute PEF increased by 7.3 L/min per year (95% CI 2.6–12.1) (efigure 3). The progression of absolute PEF stabilized between 10 and 14 years, similar to absolute FVC.

One hundred thirty-six of 298 (46%) patients with SMA type 2 whose data at latest visit were available required NIV. Of those, 98 of 256 (38%) were sitters, 28 of 46 (61%) were nonsitters, and 10 were missing sitting ability. Eight of 71 (11%) patients with SMA type 3 required NIV. For those who had started NIV, median (range) age was 5.0 (1.8–16.6) years in SMA type 2 and 15.1 (13.8–16.3) years in SMA type 3. FVC%P at start of NIV was 44% (IQR 28.5–57) in SMA type 2 (n = 55). Thirteen (24%) had FVC%P >60%; 19 (35%) had FVC%P of 40% to 60%; 14 (25%) had FVC%P of 20% to 40%; and 9 (16%) had FVC%P <20%. Table 2 provides details on NIV establishment and requirement per day.

**Table 2:**
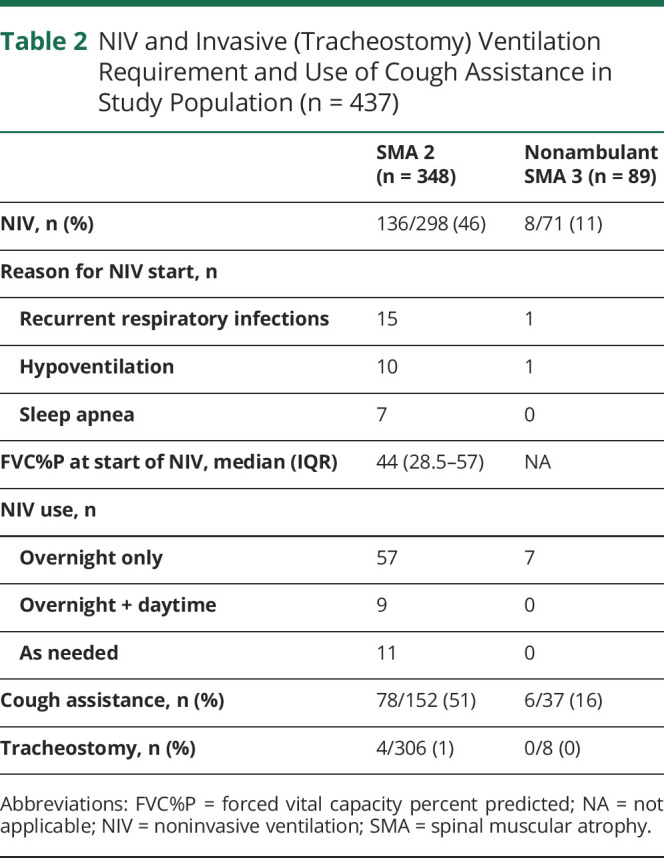
NIV and Invasive (Tracheostomy) Ventilation Requirement and Use of Cough Assistance in Study Population (n = 437)

### Correlation Between Respiratory and Motor Function

HFMS score positively correlated with FVC%P in patients with SMA type 2 (n = 76) (*r* = 0.67 *p* < 0.001) and nonambulant type 3 (n = 28) (*r* = 0.68, *p* < 0.001). RULM score positively correlated with FVC%P in SMA type 2 (n = 32) (*r* = 0.61 *p* < 0.001) and in SMA type 3 (n = 21) (*r* = 0.61, *p* < 0.01) (figure 5).

**Figure 4:**
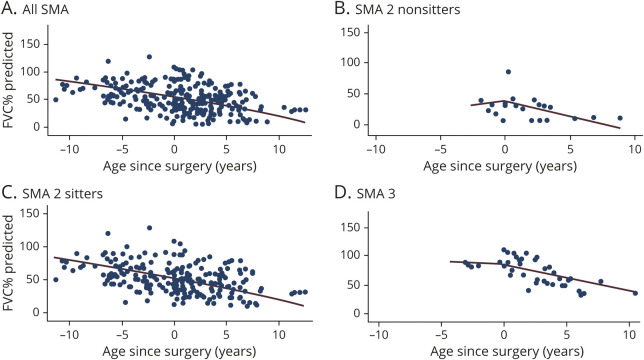
Slope of FVC%P Before and After Spinal Surgery in SMA Types 2 and 3 (A) Overall population. Forced vital capacity percent predicted (FVC%P) declined yearly by 2.7% before scoliosis surgery and declined by 3.6% afterward (*p* = 0.22). (B) Spinal muscle atrophy (SMA) type 2 nonsitters. FVC%P increased by 2.4%/y before scoliosis surgery and declined by 5.2% afterward (*p* = 0.19). (C) SMA 2 sitters. FVC%P declined yearly by 2.8% before scoliosis surgery and declined by 3.4% afterward (*p* = 0.49). (D) SMA 3 nonambulant. FVC%P declined by 1.3%/y before scoliosis surgery and declined by 4.2% afterward (*p* = 0.48).

**Figure 5:**
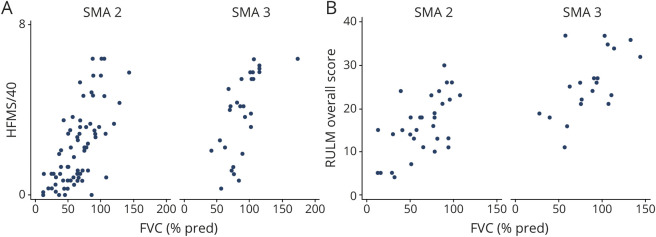
Correlation Between Respiratory and Motor Function in SMA Types 2 and 3 at First Available Visit (A) Correlation between forced vital capacity (FVC) percent predicted (FVC%P) and Hammersmith Functional Motor Scale (HFMS) score in spinal muscle atrophy (SMA) type 2 (*r* = 0.67, *p* < 0.001) and SMA type 3 nonambulant (*r* = 0.68, *p* < 0.001). (B) Correlation between FVC%P and revised Upper Limb Module (RULM) score in SMA type 2 (*r* = 0.61 *p* < 0.001) and SMA type 3 nonambulant (*r* = 0.61, *p* < 0.01).

HFMS score was available at NIV establishment in 39 patients with SMA type 2. Thirty-one (79%) had an HFMS score <10, and 24 (62%) had an HFMS score <6. HFMS score at NIV establishment in SMA type 3 and RULM score in SMA types 2 and 3 were available for only a few patients and were not analyzed.

### Respiratory Function and Scoliosis

One hundred eighty of 215 (84%) patients with SMA types 2 and 39 of 49 (80%) with nonambulant SMA type 3 whose information was available at latest visit had scoliosis. Seventy of 145 with SMA type 2 (48%) and 11 of 35 (31%) with SMA type 3 underwent scoliosis surgery. Details on the different surgical techniques, ranging from the use of growing rods in the younger children to full fixation in the older population, are shown in table 1. Median (IQR) age at surgery was 11 (8–14.4) years in SMA type 2. Sixty-five were sitters and had surgery at a median (IQR) age of 11 (8–13.2) years; 11 were nonsitters and had surgery at a median (IQR) age of 9.8 (8.2–16.1) years. Median age (IQR) at surgery in SMA type 3 was 11.7 (10.8–15.9) years (n = 16), similar to SMA type 2 (*p* = 0.12).

A worsening trend of FVC%P was observed in the whole population after spinal surgery, going from −2.7%/y to −3.6%/y, although this change was not significant (*p* = 0.22). SMA type 2 nonsitters had the largest difference in respiratory function after surgery, going from 2.4%/y to −5.2%/y. This difference was not significant (*p* = 0.19). In SMA type 2 sitters, FVC%P slope went from −2.8%/y to −3.4%/y (*p* = 0.49). In SMA type 3, it went from −1.3% to −4.2% (*p* = 0.48) (figure 4). Similarly, FVC absolute yearly slope was not different before and after surgery in SMA type 2 (overall and subtypes) and type 3.

## Discussion

In the last decade, the availability of new treatments has prompted the need for a precise understanding of the natural history of ambulant and nonambulant patients with SMA, from the most severe to the milder subtypes. Most of the emphasis has been devoted to the careful analysis of the progression of motor function because this was often the main outcome measure in clinical trials. Recently, the description of the natural history of motor function in SMA types 2 and 3 has identified that, in the early years after diagnosis, an improvement in SMA type 2 can occur, followed by a subsequent decline.^26^ This information is essential when planning clinical trials and assessing the efficacy of intervention in a broad population. However, very few studies have focused on respiratory function, and none examined the correlation between respiratory and motor function in a real-world broad population of intermediate SMA.

Recent long-term data from patients with SMA types 2 and 3 enrolled in the nusinersen clinical trials showed remarkable results on motor function (improvement of HFMSE and RULM score)^16^ but with incomplete data on respiratory outcomes. As nusinersen has become commercially available for all types of SMA in some countries, real-world data on motor and respiratory function are becoming available.

In this scenario, the phenotyping of motor and respiratory function in SMA subtypes and age range is crucial to establish the actual efficacy of nusinersen and other new treatments.

Our work adds long-term respiratory data from a large international cohort (n = 437) of patients with SMA type 2 and nonambulant type 3 followed up over 8 years. Similar to motor function,^26,27^ the decline of FVC%P in SMA type 2 and type 3 followed different trajectories across age ranges. In both subtypes, FVC%P declined more steeply from 5 to 13 years (≈4%/y in SMA type 2 and ≈3%/y in type 3), followed by a slower annual progression after 13 years of age (1% in SMA type 2 and 0.9% in type 3). However, in type 3, the FVC%P decline was more obvious after the age of 8 years (rather than 5 years as observed in type 2), with a pattern similar to the slopes of the 6-minute walk test. The rate of decline significantly changed after 13 years in both SMA types. The FVC%P declined to a stable level after 13 years and remained higher in type 3 (≈66%) than in type 2 (41% sitters, 32% nonsitters). It is of interest that although nonsitters generally have a more severe phenotype, there was no significant difference in the rate of decline between sitters and nonsitters.

The annual decline of FVC%P had been reported as being 2.9% in 79 children and young adults with SMA type 2 and type 3 over 36 months. Patients with SMA type 2 declined faster than those with type 3; however, the relatively small sample size did not allow further conclusions.^13^ In a separate retrospective study on 31 patients with SMA types 2 and 3 (age range 3–21 years), the median decline of the FVC%P was 7.9% in SMA type 2 and 2.8% in type 3.^14^ The results cannot be easily compared because of the different sizes of the 2 populations studied and because the previous study, as opposed to ours, also included ambulant type 3. Our considerably larger population and the longer observation period allowed the identification of age-specific trajectories of pulmonary function selectively for SMA types 2 and 3. We explored the potential use of the PEF as a surrogate of expiratory muscle function in SMA type 2. In other neuromuscular disorders such as Duchenne muscular dystrophy, PEF captured respiratory decline earlier than FVC%.^28^ In patients with SMA, our findings showed that PEF%P declined consistently with FVC%P, confirming its potential utility as a measure of pulmonary function in SMA.

According to the most recent standard of care, FVC%P <60% and FVC%P <40% are associated with the increased risk of REM-related and non–REM-related sleep disordered breathing, respectively.^4,29^ In SMA type 2, FVC%P fell below 60% at a median age of 12.8 years, and at the age of 13.4 years, 25% with SMA type 2 had FVC%P <40%. In our cohort, 39% of those with SMA type 2 had started NIV at a median FVC%P of 44% (28.5%–57%) at a median age of 5 (1.8–16.6) years. Despite >75% of patients with SMA type 3 in our cohort maintaining a FVC%P >60% at 18 years, 9% of patients with SMA type 3 required NIV at a median age of 15.1 (13.8–16.3) years. NIV was started more frequently to treat acute respiratory decompensation during chest infections. Given the relatively incomplete information on the main reason to start NIV, it was not possible to retrospectively assess whether, for example, chest infections occurred more frequently in patients not using cough assistance. Similarly, details on the frequency that NIV support was used as needed during acute decompensations only as opposed to chronic use were not available. Our results confirm that a reduced FVC%P (≈40%) and an increased number of respiratory infections are strongly associated with NIV requirement, as recently reported in a cross-sectional study of pediatric SMA types 1, 2, and 3.^30^ The revised standard-of-care guidelines promote the early adoption of cough assist devices and a proactive early respiratory workup to identify the need for NIV establishment. Our study did not contain information on whether the adoption of such pulmonary measures reduced the number of respiratory infections, an important consideration for future studies. We acknowledge, however, that the more proactive use of cough assistance and NIV in recent years could act as a confounding factor in the analysis of long-term respiratory function.

Although functional motor data were limited because some of the tools used such as the RULM became available only recently, our data suggest that FVC%P positively correlates with HFMS and RULM scores. In an attempt to translate this into clinically meaningful thresholds, we found that at the time when NIV was started, 31 of 39 with SMA type 2 had an HFMS score ≤10 and 24 had an HFMS score ≤6.

Scoliosis was a common feature of both SMA type 2 (84%) and nonambulant SMA type 3 (80%), in keeping with previous data.^31^ Median (IQR) age at surgery was 11 (8–13.3) years in SMA type 2 and 11.7 (10.8–15.9) years in SMA type 3. We evaluated whether the surgical correction of scoliosis could influence the respiratory progression. A few studies have cross-sectionally evaluated the pulmonary function before and after scoliosis with controversial results.^32–34^ We have compared the annual decline of FVC%P and absolute FVC. Their trajectories after surgery were steeper than before surgery, even though the difference was not significant (*p* = 0.22). The steep decline of pulmonary function occurring in the year after surgery possibly contributes to this negative results^35^; in addition, different surgical techniques were used in this group of patients, ranging from different types of growing rods to full spinal fusion, contributing to the heterogeneity of the outcome. Because respiratory and motor functions correlate, we confirm the data of a recent report on 17 patients with SMA types 2 and 3 suggesting that motor function (HFMSE score) significantly and permanently worsened after surgery.^36^

Patients' nutritional status expressed as BMI and respiratory function expressed as FVC correlated in both SMA types 2 and 3. This finding suggests that the regular body growth and a wider rib cage allow the expansion of the lungs and are positively associated with higher lung volumes. In patients with SMA, an adequate nutritional intake should be monitored and promoted.^37^

To the best of our knowledge, this is the largest observational study on long-term respiratory function in SMA type 2 and nonambulant type 3 reported to date. It provides data on respiratory function measures in addition to FVC%P, along with data on the correlation with motor function and the requirement for NIV. The breakdown of severity within SMA type 3 (i.e., types 3A and 3B) was beyond the purposes of the current work because we decided to exclude ambulant SMA type 3. The identification of thresholds of pulmonary function associated with clinically meaningful events such as sleep disordered breathing, use of NIV, or recurrent respiratory infections was limited by the retrospective nature of our study and missing data. While we acknowledge that the retrospective design was the main limitation of our study, our registry is systematically promoted in each of the 3 participating networks that have been contributing to real-world data collection since 2010.

These data should be confirmed in larger prospective studies, which may allow the establishment of more precise thresholds of motor function scores associated with different levels of respiratory function. The upcoming setup of a unique customized platform within iSMAc^20^ will allow more robust data collection for prospective longitudinal study of respiratory function in intermediate SMA types and the effect of new treatments.

Our work adds long-term respiratory data from a large international cohort (n = 437) of patients with SMA type 2 and nonambulant type 3 followed up over 8 years. Similar to what has been described in motor function,^26,27^ the decline of FVC%P in SMA type 2 and type 3 followed different trajectories across age ranges. FVC%P annual decline was steeper from 5 to 13 years (≈4% in SMA type 2 and ≈3% in SMA type 3), followed by a slower progression after age 13 years (1% in SMA type 2, 0.9% in type 3). However, in SMA type 3, the decline was more obvious after age 8 years (rather than age 5 years as in SMA type 2). Although nonsitters generally had a more severe phenotype, there was no significant difference in their rate of decline compared to sitters. In both SMA types 2 and 3, the motor (HFMS and RULM scores) and respiratory functions correlated positively. The data provided by this study will be important in the interpretation of the long-term real-world respiratory outcome of patients who are now being treated with disease-modifying therapies.

## Glossary

BMI: body mass index
CI: confidence interval
FVC: forced vital capacity
FVC%P: FVC percent predicted
HFMS: Hammersmith Functional Motor Scale
IQR: interquartile range
iSMAc: International SMA Consortium
NIV: noninvasive ventilation
PEF: peak expiratory flow
PEF%P: PEF percent predicted
RULM: revised Performance of Upper Limb
SMA: spinal muscular atrophy
*SMN*: survival motor neuron
ULM: Upper Limb Module
